# Impact of heat exposure during immobilization‐induced de‐training and re‐training on aerobic capacity and haemoglobin mass

**DOI:** 10.1113/EP092931

**Published:** 2026-01-06

**Authors:** Scott Cocking, Nathan Townsend, Mariem Labidi, Khouloud Mtibaa, Marine Alhammoud, Nada Nasir, Nelda Nader, Karim Khalladi, Claire Tourny, Abdulaziz Farooq, Sebastien Racinais

**Affiliations:** ^1^ Department of Sports Science Aspire Academy Doha Qatar; ^2^ College of Health and Life Sciences Hamad Bin Khalifa University Doha Qatar; ^3^ Aspetar, Orthopaedic and Sports Medicine Hospital Doha Qatar; ^4^ Faculty of Sport Sciences and Physical Education CETAPS, University of Rouen Rouen France; ^5^ Environnemental stress unit CREPS Montpellier – Font Romeu Montpellier France; ^6^ DMEM, Univ Montpellier, INRAE Montpellier France

**Keywords:** aerobic capacity, de‐training, heat acclimation, immobilization, rehabilitation

## Abstract

The aim of this work was to assess the effect of heat exposure on cardiorespiratory and haematological responses during de‐training and re‐training. Nineteen men (33.8 ± 2.7 years; 182 ± 5.7 cm, 84.4 ± 9.3 kg) completed 4 weeks of pre‐training followed by heat exposure (HEAT; *n *= 9) or control (CON; *n* = 10). Both groups then de‐trained for 2 weeks with lower‐limb immobilization followed by 2 weeks of re‐training. Cardiorespiratory fitness and total haemoglobin mass (Hb_mass_) were measured at baseline (BASE), prior to (IMMO_pre_) and following (IMMO_post_) immobilization and after a ‘return‐to‐sport’ (RTS_post_) training phase. Compared with IMMO_pre_, ${\dot{V}}_{O_{2}}$ at the gas exchange threshold (GET) was reduced (*d *= −0.53; *P *< 0.005) at IMMO_post_, whereas maximal oxygen uptake (${\dot{V}}_{O_{2}\max}$) did not change significantly (*d *= 0.14; *P* = 0.07). The reduction in GET ${\dot{V}}_{O_{2}}$ was more pronounced for HEAT than CON (*d *= −0.77; *P* = 0.001). At IMMO_post_, GET and peak power output were lower than IMMO_pre_ (*d *= −1.4, *P *< 0.005 and *d *= 0.43, *P *= 0.01, respectively), however there was no difference between groups. At RTS_post_, the ${\dot{V}}_{O_{2}}$ at GET increased again in both groups yet remained lower than IMMO_pre_ although the reduction in HEAT (*d *= −0.42; *P* = 0.43) was less than CON (*d *= −0.8; *P *= 0.05). At RTS_post_, HEAT fully recovered GET power losses compared to IMMO_post_ (*d *= 1.1; *P *= 0.001), while CON only showed a trivial change (*d *= 0.07; *P *= 0.1). No significant changes were observed in Hb_mass_, haematocrit or plasma volume throughout the study (*P* ≥ 0.763). Heat exposure did not attenuate a decline in cardiorespiratory fitness during immobilization‐induced de‐training, but could potentiate its recovery upon re‐commencement of training.

## Abstract (highlights)

- **What is the central question of this study?**
  Does a combination of passive and active heat exposure during a de‐training phase induced by single lower limb immobilization attenuate expected declines in aerobic capacity?
- **What is the main finding and its importance?**
  Passive heat exposure during 2 weeks of immobilization did not attenuate a decline in aerobic capacity compared with a placebo condition. During a subsequent 2 weeks of exercise training and heat exposure phase, there were greater increases in aerobic function compared to the placebo. Heat exposure implemented during a rehabilitation scenario is more likely to have a beneficial effect on cardiorespiratory fitness when added to the re‐training exercise phase as opposed to an immobilization‐induced de‐training phase.

## 1 INTRODUCTION

Aerobic exercise capacity depends on both cardiovascular and skeletal muscle function in humans (Richardson et al., 2000). These systems improve or decline following periods of training or detraining, respectively (Coyle et al., 1984). Detraining can be defined as the partial or complete loss of training‐induced adaptations in response to an insufficient training stimulus, which may take place within short periods of training cessation or during marked reductions in habitual physical activity levels (Mujika & Padilla, 2001). Musculoskeletal injury is a common cause of detraining in previously well‐trained individuals. For example, it takes athletes up to ∼6 months to return to sport after anterior cruciate ligament reconstruction (Zaffagnini et al., 2015), but could be significantly shorter or longer depending on the type and severity of injury (Ardern et al., 2011). The period of complete (in case of surgery) and partial unloading (during re‐training) can lead to various forms of musculoskeletal and cardiovascular deconditioning; for instance, 2–8 weeks of physical de‐conditioning decreases ${\dot{V}}_{O_{2}\max}$ by ∼4–20%, likely due to decreases in blood volume, stroke volume and cardiac output (Mujika & Padilla, 2001).

Chronic heat exposure has been well established to promote central cardiovascular adaptions that tend to increase total blood volume (Oberholzer et al., 2019; Racinais et al., 2024; Rønnestad et al., 2021). Over the past few decades, a variety of heat acclimation methods and protocols have been proposed including 30–90 min daily exposure for 1–3 weeks to elevated ambient temperatures with (active) or without (passive) exercise (Daanen et al., 2018). Repeated passive heat exposure protocols have been demonstrated to trigger a suite of beneficial cardiovascular and thermoregulatory adaptations in trained athletes (Brazaitis & Skurvydas, 2010; Racinais et al., 2017a), enhance skeletal muscle and mitochondrial function in healthy individuals (Hafen et al., 2018; Racinais et al., 2017b), and improve endothelial function, arterial stiffness and blood pressure in sedentary adults (Brunt et al., 2016a, 2016b),

Therefore, a potential intervention to attenuate the effect of de‐training‐induced impairments to cardiovascular function and aerobic capacity could be provided by chronic heat exposure implemented following an injury during the period of forced reduction in training load (Ihsan et al., 2019). Moreover, adding heat stress during the re‐training period may potentiate cardiovascular adaptations (Ihsan et al., 2019) thereby allowing for a lower mechanical load and risk of re‐injury during rehabilitation (Racinais et al., 2021). Even though passive heat acclimation has been established to induce plasma volume expansion (Wilson et al., 2020), recent evidence also indicates total haemoglobin mass could increase (Oberholzer et al., 2019). Thus, it has been suggested that passive heating alone could be utilized by immobilized athletes as a surrogate to exercise to either maintain cardiovascular fitness or attenuate a decline resulting from de‐training (Ihsan et al., 2019). No studies have examined the effect of passive heat acclimation on cardiorespiratory fitness and blood volume during a period de‐training though.

The aim of this study was to investigate the effect of whole‐body repeated heat stress during 2 weeks of lower limb immobilization (passive heat only exposure) followed by 2 weeks of exercise training (passive + active heat exposure) on aerobic capacity, cardio‐respiratory responses, and total blood volume. It was hypothesized that repeated heat exposures may partly counteract the negative effect of detraining and preserve cardiorespiratory fitness level of participants during a simulated ‘return‐to‐sport’ rehabilitation phase.

## 2 METHODS

### 2.1 Participants

This study was part of a larger project examining skeletal muscle adaptation (Labidi et al., 2024), for which a non‐inferiority sample size calculation (G‐Power, software version 3.1.9.6) was conducted based on the maximal isometric force effect size reported from Racinais et al. (2017b). The sample size calculation indicated a minimum of nine participants were required per group with significance and power set at 0.05 and 80%, respectively. Initially 22 participants were recruited, whilst 19 completed the entire 8‐week experimental protocol. All participants (mean ± SD: 33.8 ± 2.7 years, 182 ± 5.7 cm, 84.4 ± 9.3 kg) were healthy, had a sport background, no neuromuscular disorders and no ankle injuries. All were former competitive athletes, primarily in athletics, now working as fitness coaches. The project was ethically approved for males only since a higher thrombosis risk has been reported with lower limb immobilization in females using oestrogen‐containing contraceptives or hormone therapies (Keenan et al., 2018). Participants were informed about the possible risks and signed informed consent before commencement of the study. The experimental procedures were conducted in accordance with the *Declaration of Helsinki* concerning research on human participants (Ethical Principles for Medical Research Involving Human Subjects) and approved by the hospital scientific committee (no. ASC/0000205/ak) and the Aspire Zone Foundation institutional review board (no. F202007002).

### 2.2 Experimental design

**FIGURE 1:**
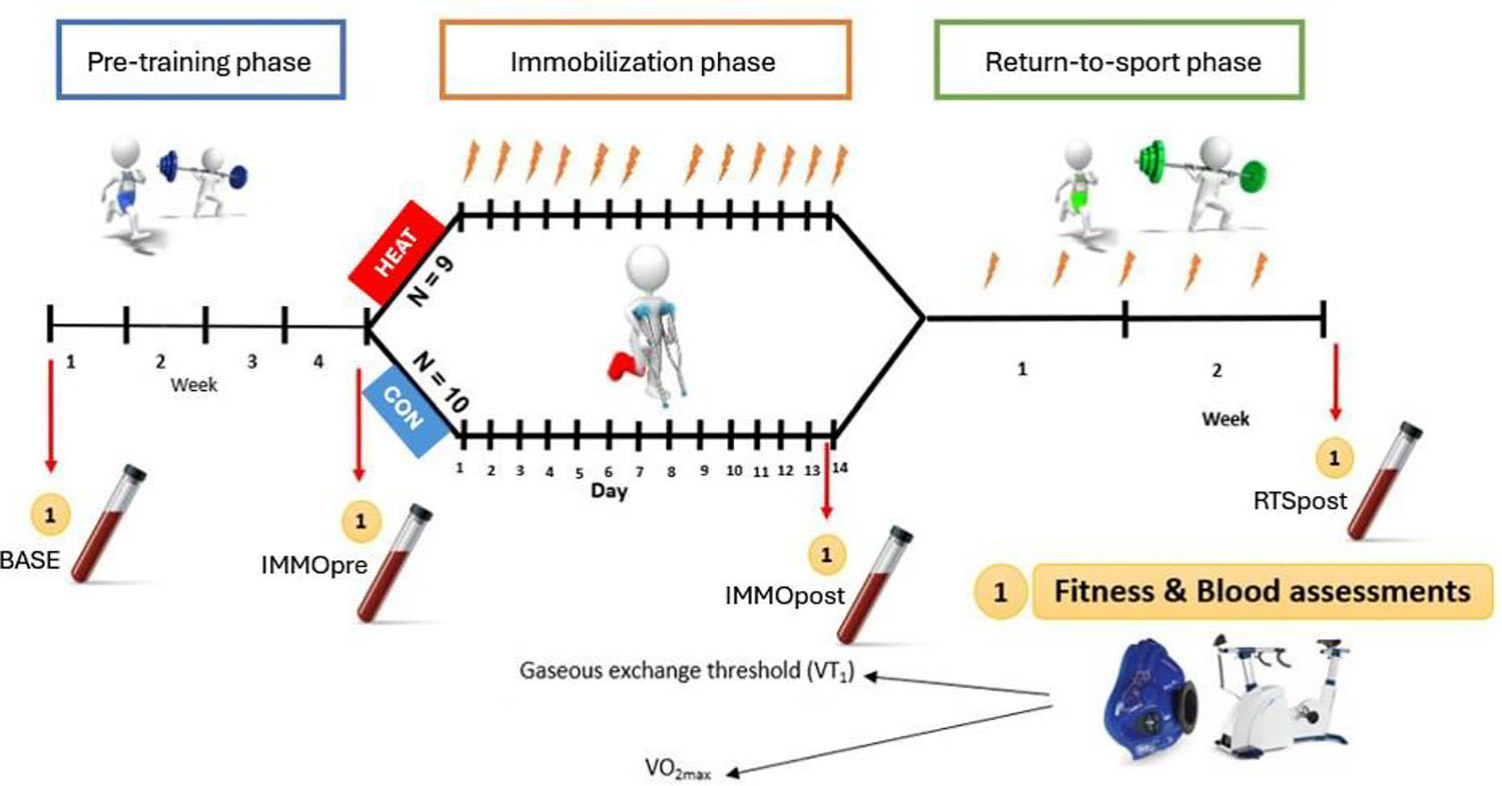
Outline of experimental protocol. All participants completed a standardized 4‐week ‘wash in’ pre‐training phase and were then randomly divided into intervention (HEAT) and control (CON) groups. Each group underwent 2 weeks of single‐leg immobilization in an ankle boot with cessation of all exercise training. Thereafter, all participants completed a 2‐week ‘return‐to‐sport’ (RTS) rehabilitation training phase. Incremental exercise testing and haematological measures were taken before the initial pre‐training phase (BASE), immediately prior to and following the immobilization phase (IMMO_pre_ and IMMO_post_), and upon completion of the final 2 weeks of RTS phase (RTS_post_).

The experimental design for this study was a randomized controlled trial. One week after a familiarization session, participants completed in the following order: (1) 4 weeks of supervised training, then (2) 2 weeks of single‐leg immobilization and cessation of all exercise, and lastly (3) 2 weeks of supervised re‐training (Figure 1). Following the initial 4‐week supervised training period, participants were randomly separated into the experimental intervention group who underwent a combination of passive and active heat exposure sessions (HEAT; *n* = 9), and a control group (CON; *n* = 10). Participants in the HEAT group initially sat for 10 min at 50°C and 50% relative humidity (RH), thereafter adjusted to 48°C and 50% humidity for a further 50 min. This protocol has been previously demonstrated to elevate body core temperature of sufficient magnitude and duration to promote significant elevations in core temperature and measurable heat adaptation (Racinais et al., 2017a, 2017b). Participants in the CON group sat for 60 min in a similar sized normobaric hypoxia chamber at 24°C, 40% RH and 200 m simulated altitude, but were told the altitude was equivalent to 3000 m. All participants were informed the purpose of the study was to compare heat versus altitude on physiological outcomes to generate a placebo control condition. At commencement of the immobilization phase, both groups were placed in an ankle boot and instructed to use crutches throughout, and complete no exercise training whatsoever. After the immobilization phase was completed, the ankle boot was removed and participants commenced supervised training sessions again. Data collection of dependent variables was conducted at baseline (BASE), after the initial 4‐week pre‐training phase and immediately prior to immobilization (IMMO_pre_), after 2 weeks of immobilization (IMMO_post_), and following the 2‐week ‘return to sport’ training phase (RTS_post_).

#### 2.2.1 Pre‐training phase

To ensure a standardized training status prior to the immobilization phase, all participants completed a 4‐week ‘wash‐in’ training regime, consisting of three resistance exercise sessions (Sunday, Tuesday and Thursday) and two aerobic training sessions (Monday and Wednesday) per week. All of these sessions were performed in a temperate environment (∼22°C) at sea level. The resistance training sessions focused on lower limb exercises normalized to bodyweight (BW) and/or repetition maximum (RM) including seated leg press (3 sets × 10–14 reps @ 50–75% BW), standing calf raises (3 × 14–20 @ BW), seated calf raise (3 × 14–18 @ 50% BW and 6 × 10 @ RM), squat jumps (3 × 12–20 @ BW), reverse lunges (3 × 10 @ 115% BW), and Nordic hamstring curls (2–4 × 6 @ BW). The aerobic training was completed on a cycle ergometer and consisted of three different sessions (rotated sequential order): 40–60 min continuous steady‐state @ 1.3–1.9 W/kg BW; a high‐intensity interval session = 60 min total including 16 reps of 1 min @ 2.5 W/kg with 1 min recovery @ 1.1 W/kg; and a sprint interval session = 60 min total including 8 reps of 30 s @ all‐out max effort with 4 min 30 s passive recovery.

#### 2.2.2 Immobilization phase

The left ankle of each participant was immobilized at an angle of 90° for 14 consecutive days using a therapeutic boot. The participants received instructions for using the therapeutic boots and medical arm crutches. They were allowed to ambulate freely but were instructed to not engage in any weight‐bearing activity with the immobilized ankle, and to cease any exercise training. All participants reported to the laboratory 6 days per week over the immobilization period to receive the experimental intervention.

#### 2.2.3 Return‐to‐sport (RTS) phase

Following the immobilization period, the ankle boot was removed, and participants commenced a supervised exercise programme for a further 2 weeks, consisting of three sessions of aerobic conditioning (conducted in HEAT or CON conditions) and two sessions of neuromuscular training per week (one of which was conducted in HEAT or CON) designed to mimic a typical rehabilitation programme following lower limb injury. The aerobic conditioning sessions were similar to the pre‐training bicycle workouts, but slightly shorter and easier (40 min @ 1.2–1.9 W/kg). The neuromuscular sessions involved a range of bodyweight only exercises with emphasis on motor coordination as opposed to strength alone (e.g. bosu lunges, Bulgarian squats, side stepping, etc.). The HEAT group performed the three aerobic sessions in the heat chamber set at 35°C and 60% humidity, while the CON group performed those sessions in an altitude room at 200 m of altitude, 24°C and 40% RH. The non‐intervention neuromuscular training sessions were performed in a temperate environment (∼22°C) at sea level for both groups.

### 2.3 Experimental protocols and measures

#### 2.3.1 Incremental exercise testing

Each participant performed incremental cycling tests on an electromagnetically braked cycle ergometer (Excalibur, Lode, Groningen, The Netherlands), adjusted to each participant's specifications and with the feet securely strapped in the pedals. The test commenced with a 7 min warm‐up consisting of 2 min at 50 W, then 3 min at 1.5 W/kg (to determine mean response time), and then 2 min at 50 W. Thereafter, the ramp phase of the incremental cycling test commenced and increased by 25 W/min until volitional exhaustion. Participants were instructed to cycle at a preferred cadence (at least 80 rpm) and encouraged to maintain this throughout the incremental protocol. Oxygen uptake was measured continuously using an online breath‐by‐breath cardiopulmonary system (Innocor™, Rome, Italy). Heart rate (HR) was continuously monitored using a telemetric chest strap device (Suunto, Vantaa, Finland) during incremental exercise testing whilst rating of perceived exertion (RPE) using a 6–20 scale (Borg, 1982) was recorded every 2 min until volitional exhaustion.

All breath‐by‐breath ${\dot{V}}_{O_{2}}$ data were initially filtered for aberrant breathing artefacts (e.g., coughing, swallowing, sighing, etc.) that lay more than 3 SD outside the local breath‐by‐breath mean and then transformed into 5 s bin averages. The gaseous exchange threshold (GET) was estimated as the metabolic rate (i.e. ${\dot{V}}_{O_{2}}$) corresponding to the first nonlinear breakpoints of the ventilatory equivalent for oxygen (${\dot{V}}_{E}/{\dot{V}}_{O_{2}}$) and end‐tidal O_2_ ($P_{\mathrm{ET}O_{2}}$) curves, whilst the ventilatory equivalent for carbon dioxide (${\dot{V}}_{E}/{\dot{V}}_{CO_{2}}$) and end‐tidal CO_2_ ($P_{\mathrm{ETC}O_{2}}$) remained stable (Whipp et al., 1981). Before beginning ramp‐incremental exercise, a step‐transition protocol was utilized to determine mean response time (Iannetta et al., 2019). Briefly, this approach involves measurement of the steady‐state oxygen uptake (${\dot{V}}_{O_{2}}$) at a constant work rate estimated to reside within the moderate intensity domain in the current sample cohort (1.5 W/kg), prior to commencement of the ramp incremental phase. Thereafter, the power output during the ramp phase coinciding with this steady‐state ${\dot{V}}_{O_{2}}$ was determined and the discrepancy is converted into a duration (in seconds) based on the ramp rate (25 W/min) and represents the mean response time. Maximal oxygen uptake (${\dot{V}}_{O_{2}\max}$) was determined as the highest 30 s moving average and peak power output (PPO) was taken as the maximal work rate achieved during the ramp incremental test. HR values corresponding to GET, and ${\dot{V}}_{O_{2}\max}$ time points were determined from continuously recorded (1 Hz) data, and the RPE ratings most closely associated with GET and ${\dot{V}}_{O_{2}\max}$ time points were used for subsequent statistical analysis.

#### 2.3.2 Haemoglobin mass (Hb_mass_)

Resting blood samples were collected on arrival, before any exercise or heat exposure. The blood was collected in K_2_EDTA Vacutainers (Becton Dickinson, Franklin Lakes, NJ, USA) and analysed for complete blood count (CBC) on a Sysmex XT 2000i haematology analyser (Sysmex, Norderstedt, Germany). The haematocrit (Hct) values were used to estimate the relative plasma volume (PV) changes using the formula previously described (Van Beaumont, 1972). Moreover, total haemoglobin mass (Hb_mass_) was assessed using the optimized carbon monoxide (CO) rebreathing procedure described by Schmidt & Prommer (2005). This comprised of inspiring a bolus of 99.5% pure CO (Buzwair, Doha, Qatar) in a dose of 1 mL/kg of body mass that was rebreathed for 2 min. A CO sensor (Draeger PAC7000, Draeger, Luebeck, Germany) was held in proximity to the rebreathing apparatus throughout the test to check for leaks. Fingertip capillary blood samples (200 µL) were analysed in quintuple for percentage carboxyhaemoglobin (%HbCO) and Hct using a spectrophotometer (ABL90 Hemoximeter, Radiometer, Copenhagen, Denmark) before and 7 min after commencing rebreathing. The CO rebreathing procedure provides an accurate and reliable method of measuring Hb_mass_ with a typical error of ∼1.4% in our laboratory.

### 2.4 Statistical analysis

Linear mixed models (LMM) were used with SPSS V.21.0 statistical software (IBM Corp., Armonk, NY, USA) to describe time‐related changes in all parameters. Group (HEAT, CON) was added as a between‐subjects fixed effect, and time (BASE, IMMO_pre_, IMMO_post_, RTS_post_) was included as a repeated measures factor. We employed the unstructured covariance matrix to accurately capture the within‐subject correlations across different time points. In the event of significant main effects, *post hoc* pairwise comparisons were made after adjusting for multiple comparisons using the Bonferroni correction. Cardiorespiratory and power output data are presented as mean differences (95% confidence intervals), and Cohen's *d* for effect size. Effects sizes are defined as trivial (<0.2), small (0.2–0.49), moderate (0.50–0.79) and large (≥0.80) according to Cohen (2013). Tables are presented as means ± standard error (SE) data. Statistical significance was established at *P* < 0.05.

## 3 RESULTS

**FIGURE 2:**
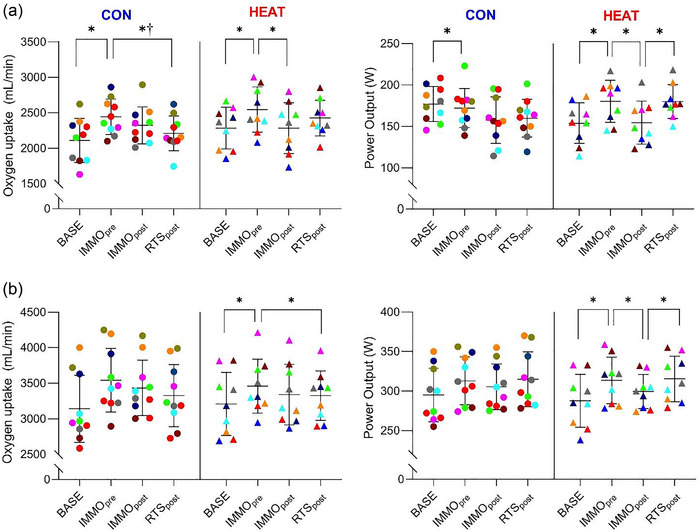
Oxygen uptake (left) and power output (right) measured at gas exchange threshold (GET) (a), and maximal oxygen uptake (${\dot{V}}_{O_{2}\max}$) (b) during the ramp incremental test. *Effect of time; † a difference between groups for the time comparison indicated (i.e. IMMO_pre_ to RTS_post_ in (a)). No markings for control indicate similar responses between conditions.

None of the measured variables showed a main effect of group (all *P* ≥ 0.05), but all showed an effect of time, and there were several group × time interactions. All ${\dot{V}}_{O_{2}}$ and power data can be seen in Figure 2 whist HR and RPE data are presented in Table 1. We observed no time, condition or interaction effects for HR at any intensity (*P* > 0.05). There were no condition or time × condition interaction effects for peak power output (*P* > 0.05).

**TABLE 1:** Heart rate (HR) and rating of perceived exertion (RPE: 6–20 scale) at the gas exchange threshold (GET) and maximal oxygen uptake (${\dot{V}}_{O_{2}\max}$) across all time points.

	CON				HEAT			
	BASE	IMMO_pre_	IMMO_post_	RTS_post_	BASE	IMMO_pre_	IMMO_post_	RTS_post_
HR: GET	144 ± 5	148 ± 3	146 ± 5	146 ± 5	152 ± 6	155 ± 3	146 ± 5	155 ± 5
HR: ${\dot{V}}_{O_{2}\max}$	183 ± 4	184 ± 3	187 ± 3	185 ± 3	182 ± 4	182 ± 3	183 ± 3	186 ± 3
RPE: GET	13.1 ± 0.5	13.3 ± 0.8	13.1 ± 0.6	12.8 ± 0.6	14 ± 0.6	13.6 ± 0.9	12.9 ± 0.6	13.9 ± 0.6
RPE: ${\dot{V}}_{O_{2}\max}$	18.4 ± 0.4	17.7 ± 0.6	18.0 ± 0.6	17.4 ± 0.4	18.0 ± 0.4	17.6 ± 0.6	17.9 ± 0.6	17.1 ± 0.4

There were no significant within groups or group × time interaction effects.

### 3.1 Pre‐training phase

From BASE to IMMO_pre_, the ${\dot{V}}_{O_{2}}$ and power at GET, PPO and ${\dot{V}}_{O_{2}\max}$ all increased significantly (all *P* < 0.001; Figure 2). There were also significant increases from BASE to IMMO_pre_ in Hb_mass_ [+27 g (95% CI: −0.3, 54 g); *P* = 0.052] and HCT [+0.8% (0.03, 1.6%); *P* = 0.041], whereas a decrease in PV [−3.7% (−7.4, 0.01%); *P* = 0.054] occurred (Table 2).

**TABLE 2:** Haematological measures: Hb_mass_ (g), haematocrit (Hct%), and relative changes in plasma volume (PV) from BASE across all time points.

	CON				HEAT			
	BASE	IMMO_pre_	IMMO_post_	RTS_post_	BASE	IMMO_pre_	IMMO_post_	RTS_post_
Hb_mass_ (g)	852 ± 29	884 ± 28	885 ± 26	872 ± 25	934 ± 29	954 ± 28	948 ± 26	937 ± 25
Hct %	42.3 ± 0.8	43.5 ± 0.8	43.7 ± 0.8	43.8 ± 0.8	43.7 ± 0.8	44.1 ± 0.8	43.7 ± 0.7	44.0 ± 0.7
PV (% change)		−5.3 ± 1.5	−5.0 ± 1.4	−4.8 ± 1.5		−2.1 ± 1.5	−2.8 ± 1.4	−3.3 ± 1.5

There were no significant within groups or group × time interaction effects.

### 3.2 Immobilization phase

Compared to IMMO_pre_, at IMMO_post_ GET ${\dot{V}}_{O_{2}}$ was moderately reduced [−190 mL (−307, −74 mL); *d *= −0.53; *P* < 0.001] whilst ${\dot{V}}_{O_{2}\max}$ showed trivial reductions [−113 mL (−232, 5 mL); *d *= −0.14; *P* = 0.071]. The reduction in GET ${\dot{V}}_{O_{2}}$ from IMMO_pre_ to IMMO_post_ was accompanied by a significant group × time interaction (*P* = 0.02) showing moderate reductions in HEAT [−261 mL (−430, −92 mL); *d *= −0.77; *P* = 0.001] but not CON (*d *= −0.09; *P* = 0.258). At IMMO_post_ there was a large reduction in GET power [−20 W (−8, 32 W); *d *= −1.4; *P* < 0.005] and a moderate reduction in peak power output [−10 W (−19, −2 W); *d *= −0.43; *P* = 0.012] relative to IMMO_pre_, with no significant difference in peak power output between groups. The group × time interaction was not significant for GET power (*P* = 0.072), although *post hoc* analysis revealed a larger reduction in HEAT [−25 W (−42, −8 W); *d *= −1.0; *P* = 0.001], compared to CON [15 W (−31, 1 W); *d *= −0.48; *P* = 0.143].

### 3.3 Return‐to‐sport phase

At RTS_post_, the GET ${\dot{V}}_{O_{2}}$ was not significantly different from IMMO_post_ (*d *= −0.01; *P* = 1.000) but was lower than IMMO_pre_ [−176 mL (−306, −45 mL); *d *= −0.8; *P* = 0.004]. ${\dot{V}}_{O_{2}\max}$ was also similar to IMMO_post_ (*P* = 0.671), but moderately lower vs. IMMO_pre_ [−174 mL (−323, −25 mL); *d *= −0.48; *P* = 0.01]. Interaction effects (*P* = 0.026) highlighted that GET ${\dot{V}}_{O_{2}}$ in CON was largely reduced vs. IMMO_pre_ values [−233 mL (−413, −54 mL); *d *= −0.8; *P* = 0.054]; however for HEAT the ${\dot{V}}_{O_{2}}$ at GET appeared to be better maintained with only moderate reductions [−119 mL (−309, 70 mL); *d *= −0.42; *P* = 0.438].

Compared with IMMO_post_, a main effect of the RTS training phase occurred with large increases in GET power output at RTS_post_ [+14 W (2, 25 W), *d *= 1.2; *P* = 0.016] whilst PPO improved moderately [13 W (4, 21 W); *d *= 0.52; *P* = 0.001]. Simple effects analysis revealed a large increase in GET power output from IMMO_post_ to RTS_post_ in the HEAT group [+25 W (8, 43 W); *d *= 1.1; *P* = 0.001], whereas the change in CON was trivial [+3 W (−14, 18 W); *d *= 0.07; *P* = 0.112), although the group × time interaction was not significant (*P* = 0.077).

### 3.4 All time points

Neither Hb_mass,_ haematocrit nor plasma volume changed between IMMO_pre_, IMMO_post_ or RTS_post_ (all *P* ≥ 0.763). There was no group × time interaction for any haematological variable (*P* ≥ 0.355) (Table 2).

## 4 DISCUSSION

The main finding of this study was that 14 days of single‐leg immobilization induced a significant de‐training effect on ${\dot{V}}_{O_{2}}$ and power output at GET, and PPO, but contrary to our hypothesis there were larger reductions in GET power and ${\dot{V}}_{O_{2}}$ in HEAT vs. CON, whilst no differences between groups occurred for the reduction in PPO. However, following a subsequent 2‐week re‐training phase, the HEAT group more effectively recovered previous losses in ${\dot{V}}_{O_{2}}$ and power output at GET. All haematological variables remained stable throughout immobilization and rehabilitation phases with no significant differences between groups observed. These results indicate that passive HEAT exposure does not attenuate the typical deterioration in cardiorespiratory fitness that occurs with a marked reduction in training load.

### 4.1 Pre‐training

The pre‐training regime was implemented to ensure the participants were not un‐trained at commencement of the experimental phase of the study, and to reduce between‐subjects variability in training status prior to limb immobilization, since the magnitude and rate of fitness losses or muscle function decay following immobilization can be influenced by baseline fitness parameters (Bickel et al., 2011; Issurin, 2010). The pre‐training ‘wash in’ consisted of two aerobic conditioning sessions and three resistance training sessions per week, for the initial 4‐week period. Similar increases in both aerobic fitness and haematological responses were observed for both groups. Whilst Hb_mass_ and haematocrit increased after the initial 4 weeks of training in both groups, there were no increases observed in either plasma or total blood volume (Table 2). All participants trained in temperate conditions prior to limb immobilization, and therefore between‐group changes in both haematological markers and training adaptation responses were arguably not expected.

### 4.2 Immobilization phase

The 14‐day immobilization phase induced non‐significant trivial changes in ${\dot{V}}_{O_{2}\max}$, whereas GET ${\dot{V}}_{O_{2}}$, GET power and PPO were all reduced (Figure 2). Somewhat unexpectedly and contrary to our hypothesis, the HEAT group experienced a significant reduction in ${\dot{V}}_{O_{2}}$ and power output at GET power, whereas CON did not. We did not observe a between groups difference for the reduction in ${\dot{V}}_{O_{2}\max}$ or PPO during the same period though. Initially we hypothesized that heat acclimation would attenuate a decline in aerobic capacity, however, it is plausible the heat intervention protocol could have influenced the exercise hyperpnoea response at low exercise intensity, but not at higher intensities. For example, it has been shown that a similar passive heat acclimation protocol as the current study elicited an increase in the exercise hyperpnoea response to incremental exercise (Beaudin et al., 2009), and in another study which examined active heat acclimation, there were changes to cerebral autoregulation found in the heat intervention group (Fujii et al., 2015). Since the GET is partially influenced by mechanisms related to the control of breathing during exercise (Whipp, 2007), these may have elicited an earlier onset of this threshold, whereas at ${\dot{V}}_{O_{2}\max}$ mechanical constraints impose a limit maximal ventilation (Johnson et al., 1992).

More stable ${\dot{V}}_{O_{2}}$ and power output at higher intensity may not be surprising, given the observed stable Hb_mass_, Hct and blood volume responses throughout immobilization. Reductions in ${\dot{V}}_{O_{2}\max}$ are typically related to reductions in blood and plasma volume (Barbieri et al., 2024), whereas physiological responses to submaximal intensity exercise (e.g., around GET) are also influenced by peripheral factors at the level of the muscle (Poole & Jones, 2012), potentially influencing more acute detraining responses. The ability to maintain both strength and muscle mass may be important factors in regaining submaximal and maximal cycling power output during rehabilitation phases following immobilization. Recent work from our lab (Labidi et al., 2024) highlights the ability of passive heat exposure during immobilization to reduce muscle atrophy responses in gastrocnemius lateralis and soleus muscles relative to a CON condition. It is important to acknowledge that site‐dependent changes in muscle mass retention may result in different functional outcomes. For example, neural detraining responses following atrophy may contribute to strength losses differently between calf and quadricep muscle groups (Casuso et al., 2024). When contextualizing these data to the current intervention, cycling primarily involves quadriceps, so caution should be applied when comparing muscle mass retention responses at differing anatomical sites. In humans, passive heat exposure (stress) in healthy active individuals can induce beneficial systemic and localized muscle cell signalling responses (Hyldahl & Peake, 2020) leading to maintained, or even enhanced (Racinais et al., 2017b), muscle function outcomes. However, these studies did not include a pre‐training wash‐in phase, nor were participants immobilized, and thus prior training history is likely relevant here. The greater reductions in GET power and ${\dot{V}}_{O_{2}}$ responses in HEAT compared to CON following immobilization would suggest that a 2‐week immobilization period overrides any metabolic or cellular signalling responses arising from the heat stimulus that might counteract detraining responses. To add further possible explanation to current study findings, classical data highlight both cardio‐respiratory and perceptual detraining responses after 2 weeks of inactivity (Coyle et al., 1985); however, detraining kinetics can vary depending on the specific physiological system involved (Coyle et al., 1984). This might also explain why we observed reductions in peak power output but not in ${\dot{V}}_{O_{2}\max}$, as power output can be influenced, not just through oxidative adaptations, but neural strength adaptations (Vikmoen et al., 2016), which are likely compromised during disuse or immobilization in quadriceps (Casuso et al., 2024).

### 4.3 Return‐to‐sport phase

During the final rehabilitation phase, we observed a larger increase in GET ${\dot{V}}_{O_{2}}$ in HEAT vs. CON. Therefore, the HEAT group appeared to return towards IMMO_pre_ levels more effectively than CON in which GET ${\dot{V}}_{O_{2}}$ remained significantly lower. Also, GET power output was improved at RTS_post_ compared with IMMO_post_ in HEAT, but not CON. Reduced atrophy responses alongside improved morphology and function following immobilization (Labidi et al., 2024) could have influenced larger improvements in both GET power and GET ${\dot{V}}_{O_{2}}$ when rehabilitation training was resumed. This remains speculative, however, given these findings are specific to calf muscle, not quadriceps (Casuso et al., 2024). Throughout rehabilitation, aerobic conditioning sessions for both groups were conducted at close to GET intensity. The importance of training specificity has been previously noted in driving adaptive responses, often overriding any potential impact of environmental factors (Racinais et al., 2021). The increase in GET power in HEAT vs. CON suggest the endurance training sessions performed in 35°C may have positively impacted return in function at RTS_post_. Training in the heat has consistently been shown to induce plasma volume expansions and enhance cardiovascular responses to aerobic exercise (Périard et al., 2016); however, we observed no changes in blood volume, Hb_mass_ or haematocrit changes throughout immobilization or rehabilitation phases, suggesting this mechanism was not responsible for any observed between‐group differences.

Oxidative enzyme activity appears to strongly correlate to submaximal exercise performance versus maximal aerobic power (Aunola et al., 1988), and passive heating has previously been shown to promote angiogenic activity and increase mitochondrial function in skeletal muscle (Hafen et al., 2018; Kim et al., 2020). ${\dot{V}}_{O_{2}\max}$ may have been expected to return to IMMO_pre_ levels given the observed peak power output improvements. Whilst this was not found, the impact of strength training on endurance capacity is well documented (Rønnestad & Mujika, 2014). Given ${\dot{V}}_{O_{2}\max}$ did not improve following the RTS phase, this suggests that strength training, regardless of environmental condition, could have been influential in improving PPO in both groups, via enhanced fatigue resistance and neural adaptations during the incremental exercise task (Casuso et al., 2024).

### 4.4 Limitations

Our participant cohort were recreationally trained males. It has been reported that de‐training and re‐training responses can vary based on training status, and therefore whether similar results occur in higher trained athletes requires further investigation. It is possible that greater decrements in aerobic power and ${\dot{V}}_{O_{2}}$ responses would occur in highly trained individuals (Coyle et al., 1984). Secondly, whilst project logistics and costs were available to recruit *n* = 22 participants and *n* = 19 completed the study, our sample size may have been slightly underpowered, potentially risking type 2 errors. Given the intensive baseline 4‐week training block undertaken, there is also the possibility participants could still have been positively adapting; for example, the immobilization phase may have acted somewhat akin to a ‘taper’ which could have confounded the de‐training responses between individuals. Enlisting a participant cohort with stable cardiorespiratory fitness over the 4‐week pre‐training phase may have resulted in larger or more consistent reductions in both ${\dot{V}}_{O_{2}}$ and power output.

### 4.5 Conclusion

Following a 4‐week period of baseline training, 2 weeks of subsequent limb immobilization led to significant detraining responses in GET ${\dot{V}}_{O_{2}}$, GET power and ${\dot{V}}_{O_{2}\max}$. The inclusion of a passive heat stimulus was associated with a larger decline in aerobic fitness in the HEAT group vs. CON. However, following a subsequent 2‐week return to sport phase involving re‐commencement of exercise training, there were larger improvements in submaximal exercise capacity in the HEAT group compared with CON. In summary, passive heat exposure did not attenuate a de‐training‐induced decline in aerobic capacity during immobilization, whereas when heat exposure was combined with exercise, this was associated with greater recovery of cardiorespiratory fitness than the CON group towards values measured following 4 weeks of pre‐training.

## AUTHOR CONTRIBUTIONS

Conception of design of the work: Scott Cocking, Nathan Townsend, Mariem Labidi, Khouloud Mtibaa and Sebastien Racinais. Analysis or interpretation of the data for the work: Scott Cocking, Nathan Townsend, Sebastien Racinais. Drafting of the work or revising it critically for important intellectual content: Scott Cocking, Nathan Townsend and Sebastien Racinais. All authors approved the final version of the manuscript and agree to be accountable for all aspects of the work in ensuring that questions related to the accuracy or integrity of any part of the work are appropriately investigated and resolved. All persons designated as authors qualify for authorship, and all those who qualify for authorship are listed.

## CONFLICT OF INTEREST

The authors declare they have no conflicts of interest.
